# New-onset Graves’ disease following SARS-CoV-2 vaccination: a case report

**DOI:** 10.1530/ETJ-22-0049

**Published:** 2022-06-29

**Authors:** Ringo Manta, Charlotte Martin, Vinciane Muls, Kris G Poppe

**Affiliations:** 1 Department of Nuclear Medicine, CHU Saint Pierre, Université Libre de Bruxelles (ULB), Brussels, Belgium; 2 Department of Infectious Diseases, CHU Saint Pierre, Université Libre de Bruxelles (ULB), Brussels, Belgium; 3 Department of Gastroenterology and Endoscopy, CHU Saint-Pierre, University Libre de Bruxelles (ULB), Brussels, Belgium; 4 Department of Endocrinology, CHU Saint Pierre, Université Libre de Bruxelles (ULB), Brussels, Belgium

**Keywords:** Graves’ disease, SARS-CoV-2 vaccine, adverse vaccine reactions

## Abstract

A 22-year-old male with a history of ulcerative colitis and nephrotic syndrome treated with immunomodulatory agents including vedolizumab and mycophenolic acid developed hyperthyroidism 2 weeks following the first administration of BNT162b2 vaccine (Pfizer-BioNTech COVID-19 vaccine). Graves’ disease (GD) was diagnosed based on the elevated thyrotropin-receptor antibody, thyroid scintigraphy and ultrasound. To this day, four cases of new-onset GD following SARS-CoV-2 vaccine were reported in patients with no previous history of thyroid disease. Two cases of recurrence of GD following SARS-CoV-2 vaccine were also reported. Although the underlying mechanisms of vaccine-induced autoimmunity remain to be clarified, there is a rationale for the association between SARS-CoV-2 vaccination and the development of Th1-mediated diseases, at least in predisposed individuals. The BNT162b2 vaccine could be a trigger for GD in some patients. However, the benefit/risk ratio remains by far in favour of SARS-CoV-2 vaccination considering the potentially higher risk of severe infection in these patients.

## Established facts

- SARS-CoV-2 can be complicated by different types of autoimmune thyroiditis.
- Few cases of Graves’ disease (GD) following SARS-CoV-2 (vaccination) have been reported.

## Novel insights

- A previous episode of SARS-CoV-2 with subacute thyroiditis and temporally hypothyroidism does not exclude GD later on.
- BNT162b2 vaccine can boost and interfere with the immune system that might lead to GD, even in patients under immunomodulatory treatment.

## Introduction

The severe acute respiratory syndrome coronavirus 2 (SARS-CoV-2) pandemic has led to accelerated development of vaccines against coronavirus disease 19 (COVID-19). Several candidate vaccines were reported by the end of 2020, including mRNA vaccines BNT162b2 (Pfizer-BioNTech COVID-19 vaccine) and mRNA-1273 (Moderna COVID-19 vaccine), delivered in a lipid nanoparticle and expressing a full-length spike glycoprotein of SARS-CoV-2 (1). The BNT162b2 vaccine was approved by European Medicine Agency and became available in December 2020. The estimated vaccine efficacy in preventing symptomatic SARS-CoV-2 infection at least 14 days following the second injection was 95% (2). Most of the reported adverse effects were minor and did not include thyroid disorders (1).

## Case report

A 22-year-old male was referred to our outpatient endocrinology clinic. The patient had a history of ulcerative colitis (UC) and nephrotic syndrome diagnosed in 2018 and 2020, respectively. He was treated with vedolizumab, oral budesonide and mycophenolic acid. In April 2020, the patient presented a mild COVID-19 and received hydroxychloroquine. Concomitantly, he developed transient hypothyroidism with negative thyroid peroxidase antibody (TPOAb) and thyrotropin-receptor antibody (TRAb). The patient also did not complain of neck pain, suggesting atypical thyroiditis as described by Muller *et al.* (3). Thereafter, bimonthly follow-up of thyroid function tests was performed. Notably, thyrotropin level was normal on 18 March 2021. On March 27, the patient received the first dose of the BNT162b2 vaccine. Two weeks later, laboratory results showed elevated levels of free thyroxine and free triiodothyronine levels along with low thyrotropin levels. Complete blood count and electrolytes were otherwise normal, as were tests of coagulation and renal function tests. Thyroid function tests were repeated 2 days and 2 weeks later, confirming hyperthyroidism. Assessment for autoimmune thyroid disease was performed in late April, showing elevated levels of TRAb and thyroglobulin with normal levels of antithyroglobulin antibodies. Laboratory test results are shown in Table 1.

**Table 1:** Laboratory test results of the patient.

Laboratory analysis	Reference range	Year 2020						Year 2021				
		March 23	April 7	April 9	June 22	August 5	December 7	February 2	March 18^a^	April 12	April 14	April 27
Thyrotropin (mIU/L)	0.27–4.20	5.42	7.36	2.86	1.35	3.21	2/29	1.84	2.76	0.02	<0.01	<0.01
Free T_4_ (pmol/L)	12.0–22.0	11.7	9.1	7.5	15.5	17.7	16.5	16.8		27.3	31.2	40.9
Free T_3_ (pmol/L)	3.10–6.80						4.96			10.9	13.2	17.7
Thyroglobulin (ug/L)	<77.0										83.7	103
Thyroglobulin (kIU/L)	<115										43	65
TPOAb (kIU/L)	<34			<15								
TRAb (IU/L)	<0.55			<0.1								3.76
PCR SARS-CoV-2			Positive	Positive								
SARS-CoV-2 IgG (AU/mL)	<13									704		

^a^The first dose of BNT162b2 (Pfizer-BioNTech) was administered on 27 March 2021.

T4, thyroxine; T3, triiodothyronine; TPOAb, thyroid peroxidase antibodies; TRAb, thyrotropin receptor antibodies.

Clinical evaluation showed subtle tremors in both upper extremities. The patient did not report palpitation, heat intolerance, emotional lability, diarrhea or weight loss. The blood pressure was 120/85 mmHg, the pulse was 85 b.p.m, the weight was 57.2 kg and the height was 172 cm. Heart sounds were normal and chest sounds were clear. There was no goiter or signs of eye involvement.

Neck ultrasonography showed heterogeneous aspect of the thyroid parenchyma associated with a marked, diffuse and bilateral intraparenchymal hypervascularization. On pulsed Doppler, circulation speed was increased in the thyroid arteries. Thyroid scintigraphy obtained 20 min after the i.v. injection of 185 mBq of technetium-99m sodium pertechnetate revealed an increased uptake with homogeneous distribution of the tracer. No thyroid nodule was found on both ultrasonography and scintigraphy.

The patient was diagnosed with Graves’ disease (GD). Treatment with methimazole 20 mg daily was initiated in early May 2021. The patient developed a mild hypothyroidism 6 weeks later. Thyroid function test then progressively improved after a thyroid hormone replacement treatment was added.

## Discussion

We report a case of new-onset GD 2 weeks following the first administration of BNT162b2 vaccine in a patient under immunomodulating treatment. Normal thyroid function tests were documented a few days prior to the administration of the vaccine. Although TRAb was not obtained just before the administration of the vaccine, TRAb was negative in April 2020 as part of the workup of transient hypothyroidism developed in the course of COVID-19. To our knowledge, four cases of new-onset GD following SARS-CoV-2 vaccine were reported in patients with no previous history of thyroid disease (4, 5, 6). Moreover, two cases of recurrence of GD following SARS-CoV-2 vaccine were also reported (4, 7). Out of these five cases, four patients received BNT162b2 and one patient received ChAdOx1 nCoV-19 vaccine. Very few cases of new-onset GD following other vaccines were reported, including six confirmed cases of GD following the administration of quadrivalent human papillomavirus (HPV). However, the association between HPV vaccine and the onset of GD remains unclear (8). An isolated case of GD following H1N1 vaccination was also reported (9). Furthermore, COVID-19 itself has been suggested as a precipitating factor for GD, although the precise pathophysiological mechanism remains unclear (10). In the present case, this hypothesis is unlikely considering that TRAb and TPOAb were negative in April 2020 and thyrotropin level was normal until 2 weeks prior to vaccination.

Vaccine-induced autoimmunity has been previously described, notably for cases of Guillain Barré Syndrome and narcolepsy following influenza vaccines. Although the underlying mechanisms of vaccine-induced autoimmunity are poorly known, the main two suggested mechanisms were cross-reactivity between antigens and effect of adjuvant (11). The latter does not apply to this case as the current SARS-CoV-2 vaccines available do not contain adjuvants (1). However, SARS-CoV-2 vaccines were intentionally designed to promote a Th1 immune response in order to avoid potential vaccine-enhanced diseases that were previously described with vaccines against other coronaviruses (12). GD itself is characterized by a Th1 response with a high number of Th1 CD4 cells and interferon secretion (13). There is therefore a rationale for the association between SARS-CoV-2 vaccination and the development of Th1-mediated diseases, at least in predisposed individuals.

Our patient is known for UC and nephrotic syndrome treated with immunomodulatory agents for more than a year. These agents are generally associated with a lower occurrence of autoimmune diseases. UC was treated with vedolizumab, a fully-humanized MAB that selectively targets α4β7 integrin expressed on circulating B and T lymphocytes. Nephrotic syndrome was treated with mycophenolic acid, an antimetabolite drug inhibiting the inosine monophosphate dehydrogenase enzyme, thereby retarding the synthesis of purines in cells in lymphocytes. Interestingly, Struja *et al.* showed that immunomodulatory agents were suggested to significantly lower the risk for relapse of GD when added to the standard treatment. Although immunosuppressive drugs involved in the study included mycophenolic acid but not vedolizumab (14), specific data are lacking regarding the underlying vaccine immune response mechanisms on the onset of GD in a patient treated with immunomodulators. Moreover, immunomodulatory agents are generally expected to lower the immunogenicity of the vaccine (15). Furthermore, prevalence of thyroid disorders is not increased in patients with inflammatory bowel disease (16).

Specific data regarding the safety and efficacy of SARS-CoV-2 vaccines in patients with immunodeficiency or autoimmune diseases are currently lacking, as most available trials have excluded these patients. However, the benefit/risk ratio remains by far in favour of SARS-CoV-2 vaccination considering the potentially higher risk of severe infection in these patients (2). Systematic reporting of cases of GD following SARS-CoV-2 vaccination will allow for assessement of the potential relationship between between the SARS-CoV-2 vaccine and the onset of GD. Clinical surveillance of thyroid function test might be considered in post-vaccination monitoring protocols in the future.

## Conclusion

The BNT162b2 vaccine could be a trigger for GD in some patients. However, SARS-CoV-2 vaccination remains encouraged considering the potentially higher risk of severe infection in patients under immunomodulatory drugs.

## Declaration of interest

The authors declare that there is no conflict of interest that could be perceived as prejudicing the impartiality of this case report.

## Funding

This work did not receive any specific grant from any funding agency in the public, commercial or not-for-profit sector.

## Consent for publication

Informed consent has been obtained from the patient (or patient’s guardian) for the publication of the case report and accompanying images.
